# Cross-Sectional coaxial technique: optimizing specimen adequacy and safety in ultrasound-guided renal biopsy procedures

**DOI:** 10.1080/0886022X.2026.2656545

**Published:** 2026-08-18

**Authors:** Lei Huang, Xiaolin Zhan, Lin Chen, Jing Zhang, Nanyan Luo, Yangyang Wang, Dongmei Wang, Jie Liu, Lili Ren, Mian Li, Qi Yang, Haiyu Luo

**Affiliations:** ^a^ Department of Ultrasound, the First Affiliated Hospital of Anhui Medical University North District (Anhui Public Health Clinical Center), Anhui, China; ^b^ Department of Infectious Diseases, the First Affiliated Hospital of Anhui Medical University North District (Anhui Public Health Clinical Center), Anhui, China; ^c^ Department of Nephrology, the First Affiliated Hospital of Anhui Medical University North District (Anhui Public Health Clinical Center), Anhui, China; ^d^ Department of Ultrasound, Peking University Shenzhen Hospital, Guangdong, China

**Keywords:** Percutaneous renal biopsy, ultrasonography, coaxial technique, complications

## Abstract

Percutaneous renal biopsy (PRB) is the gold standard for diagnosing renal diseases. This study aimed to compare the specimen adequacy and safety of three ultrasound-guided PRB techniques: cross-sectional coaxial (Group A), longitudinal coaxial (Group B), and conventional longitudinal noncoaxial technique (Group C). This single-center retrospective study enrolled 184 adult patients who underwent PRB at Anhui Provincial Public Health Clinical Center between January 2019 and December 2025. All procedures were performed by experienced interventional radiologists. Outcomes included total glomerular yield, specimen adequacy, procedural time, and bleeding complications (perinephric hematoma, gross/microscopic hematuria). Baseline characteristics were comparable across groups. Specimen adequacy was 100% in Group A, 97.8% in Group B, and 89.1% in Group C. Group A exhibited significantly superior adequacy compared to Group C (*p* = 0.016). Group A had the shortest procedural time (1.45 ± 0.20 mins), which was significantly shorter than Group B (2.22 ± 0.35 mins) and Group C (2.61 ± 0.46 mins; all *p* < 0.001). The incidence of bleeding complications was significantly lower in Group A than in Group C (perinephric hematoma: *p* < 0.001; gross hematuria: *p* = 0.002; microscopic hematuria: *p* = 0.004). A significantly higher incidence of gross hematuria was also noted in Group A compared to Group B (*p* = 0.004). The cross-sectional coaxial technique for ultrasound-guided PRB provides better specimen adequacy, shorter procedural time, and a lower bleeding risk compared with longitudinal coaxial and conventional non-coaxial techniques. This combined approach may serve as an optimized strategy to improve PRB outcomes, although multicenter randomized trials are needed to confirm its generalizability.

## Introduction

The global incidence of chronic kidney disease is on a steady rise. Percutaneous renal biopsy (PRB) is the gold standard technique for the diagnosis and management of renal diseases. Tissue adequacy and procedural safety have improved with the guidance of ultrasound and the use of automated biopsy needles. In clinical practice, the longitudinal-sectional probe position is most commonly used, with the puncture target set at the lower pole of the kidney and usually without coaxial needle guidance [1]. The key determinant for non-focal renal biopsies is the number of glomeruli, with at least 10 glomeruli required for a specimen to be adequate [2]. Complications may arise due to the invasive nature of the procedure, and bleeding being the most common complications for non-focal renal biopsies [3,4]. This drawback has motivated the development of optimized approaches aimed at enhancing diagnostic efficacy while reducing the incidence of complications.

Real-time ultrasound-guided biopsy can be performed with either longitudinal-section or cross-section, using either coaxial and non-coaxial techniques [5]. Lower complication rate and shorter procedural time for the coaxial technique have been demonstrated by several researches [2,6]. Findings of the Shamshirgar et al. [7]. who compared the efficacy of different ultrasound probe orientations during PRB suggested an advantage of the cross-sectional probe location over the longitudinal probe location, with higher diagnostic yields and fewer complications reported in the cross-sectional group. In the latest studies, Akkakrisee et al. [8]. demonstrated that the cross-sectional probe technique was independently correlated with a higher technical success rate in renal biopsy, with no concurrent increase in complication rates. However, their studies had a small sample size, and none evaluated the efficacy of coaxial needle combination use. To our knowledge, no research has combined these two components and conducted a comparison with conventional methods.

This study aimed to compare the adequacy and safety of three PRB techniques: the cross-sectional coaxial technique with cross-sectional probe location, the longitudinal coaxial technique with longitudinal-sectional probe location, and the conventional non-coaxial technique with longitudinal-sectional probe location.

## Methods

### Study design

This is a single-center, retrospective, cross-sectional investigation approved by the Ethics Committee of Anhui Provincial Public Health Clinical Center (the First Affiliated Hospital of Anhui Medical University North District, FAH‑AMU North Dist.), approval No. PJ-XJ2025-01, Medical Technology Category. The study protocol was conducted in accordance with the Declaration of Helsinki. Informed consent was waived given the retrospective nature of this study. The electronic medical records of patients who underwent kidney biopsies performed by interventional radiologists at the Department of Ultrasound were reviewed from January 1, 2019 to December 31, 2025.

### Study population

The study included all patients who underwent real-time ultrasound-guided percutaneous kidney biopsies by interventional radiologists at the Department of Ultrasound, FAH-AMU North Dist, from January 1, 2019 to December 31, 2025. Exclusion criteria include pediatric patients (< 18 years of age), biopsy requiring computed tomographic (CT) guidance, focal renal tumor biopsy, and transplant kidney biopsy.

### Patient preparation

Complete blood count and coagulation parameters were routinely evaluated before the procedure. A pre-procedure urine sample was collected for urinalysis. Abnormal coagulation parameters were corrected prior to biopsy. Acceptable thresholds were an international normalized ratio (INR) < 1.5, platelet count > 50,000/mm³, and hemoglobin level ≥ 8 g/dL, respectively. Anticoagulant and antiplatelet medications were withheld appropriately; for instance, warfarin or aspirin was discontinued at least 5 days before the procedure.

### Biopsy procedure

All patients were admitted as inpatients before the procedure. Abnormal platelet count and coagulation parameters were corrected before the procedure. All patients were placed in the prone position. Local anesthesia with lidocaine was provided by choice of noncoaxial/coaxial technique and cross-sectional/longitudinal ultrasound probe positioning for biopsy was at the operator’s discretion. All biopsy procedures were performed by one of two radiologists trained in interventional radiology with more than 8 years of experience. All biopsies were performed or supervised by experienced interventional radiologists using ultrasound systems from Mindray et al. (Mindray Resona AR with SC7-1U linear array probe, Shenzhen, China; Hitachi Aloka ARIETTA60 with C251 convex array probe, Tokyo, Japan; Esaote MyLabTwice with CA541 convex array probe, Genoa, Italy).

Tissue samples were obtained with a 18/16-gauge automated spring-loaded biopsy device featuring a 11–22 mm core length. When the coaxial technique was adopted, a 17/15-gauge coaxial needle was matched to the 18/16-gauge automated core biopsy needle respectively. The kidney with better sonographic visualization was selected as the biopsy target based on the operator’s procedural preference. The choice of biopsy needle gauge was made according to operator preference or the available stock of consumables.

Before any one of the three PRB techniques, the kidney was always evaluated from multiple views to identify the optimal target biopsy site and design the appropriate needle trajectory. After the target is confirmed, the ultrasound probe was fixed in a predefined orientation (transverse or longitudinal plane), and real-time needle guidance was performed using the “in-plane technique”. Occasionally, the position of the coaxial needle or biopsy needle could be verified in additional views for safety purposes. However, such cross-checking does not change the pre-determined needle direction or trajectory.

### Cross-sectional coaxial technique

**Figure 1.**
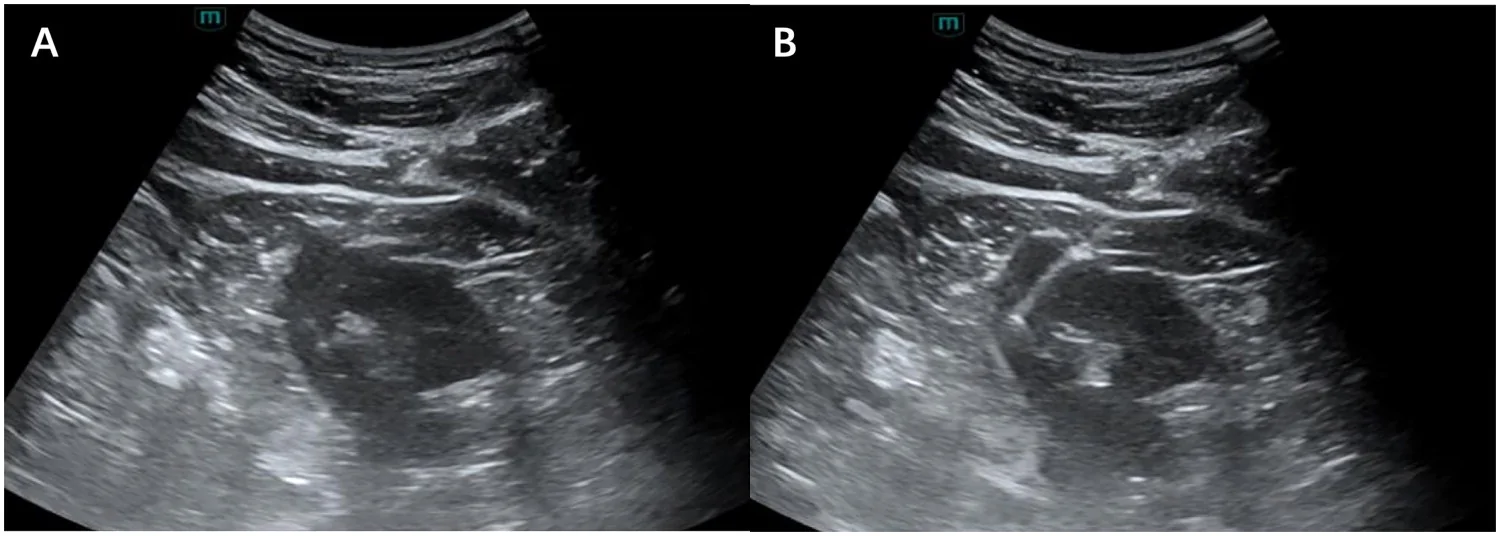
Cross-sectional coaxial technique. The middle third of the renal cortex is real-time monitored with a cross-sectional probe location. After the inner trocar stylet of the coaxial needle was withdrawn, the outer sheath of the coaxial needle was then gently pressed against the renal capsule to create a slight indentation (A), and then, the automated biopsy needle is inserted for tissue sampling (B).

For the coaxial technique with cross-sectional probe orientation, ultrasonographic localization was performed by positioning the probe in the axial plane to identify an optimal biopsy site, typically at the mid-to-lower renal pole. Under real-time ultrasound guidance, a 17- or 15-gauge coaxial needle was advanced toward the kidney with the trajectory directed away from the renal hilum. After puncturing to a depth of approximately 0.5-1 cm external to the renal capsule, the inner trocar stylet of the coaxial needle was withdrawn. The outer sheath of the coaxial needle was then gently pressed against the renal capsule to create a slight indentation, followed by the rapid insertion of an 18- or 16-gauge automated core biopsy needle (matched to the 17- or 15-gauge coaxial system, respectively) into the sheath lumen. Prior to needle advancement and activation, the patient was instructed to suspend respiration; the biopsy needle was fired immediately as its tip penetrated the subcapsular renal cortex to acquire a tissue specimen (Figure 1).

### Longitudinal coaxial technique

**Figure 2.**
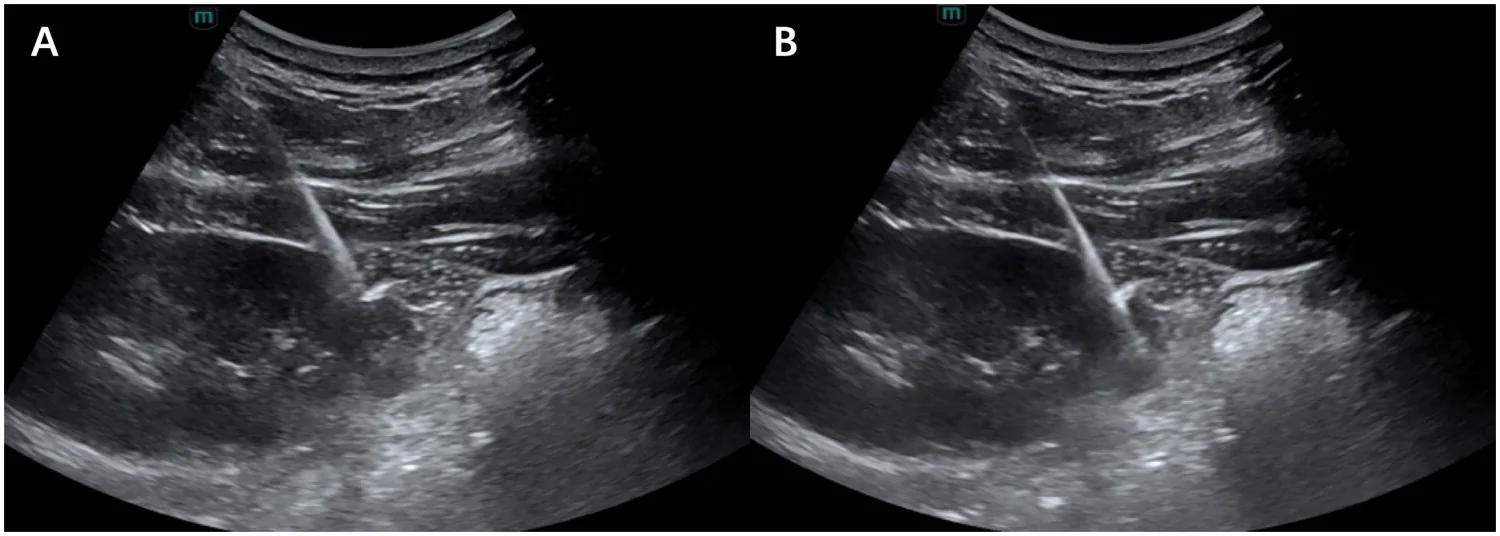
Longitudinal coaxial technique. The lower pole of the kidney is real-time monitored with a longitudinal-sectional probe location. After the inner trocar stylet of the coaxial needle was withdrawn, the outer sheath of the coaxial needle was then gently pressed against the renal capsule to create a slight indentation (A), and then the automated biopsy needle is inserted for tissue sampling (B).

For the coaxial technique with longitudinal-sectional probe location, ultrasonography was conducted by placing the probe in the sagittal position, with biopsy performed at the renal lower pole. All subsequent procedural steps were identical to those of the cross-sectional coaxial technique. Under real-time ultrasound guidance, a 17- or 15-gauge coaxial needle was advanced toward the kidney away from the hilum. After puncturing to a depth of approximately 0.5–1 cm outside the renal capsule, the inner trocar stylet of the coaxial needle was withdrawn, and the outer sheath of the coaxial needle was gently pressed against the renal capsule at the lower pole to create a slight indentation. An 18- or 16-gauge automated core biopsy needle (corresponding to the 17- and 15-gauge coaxial systems, respectively) was then rapidly advanced into the sheath lumen. The patient was instructed to hold their breath; firing was performed immediately upon penetration of the subcapsular renal cortex by the needle tip to obtain a tissue sample (Figure 2).

### Noncoaxial technique

**Figure 3.**
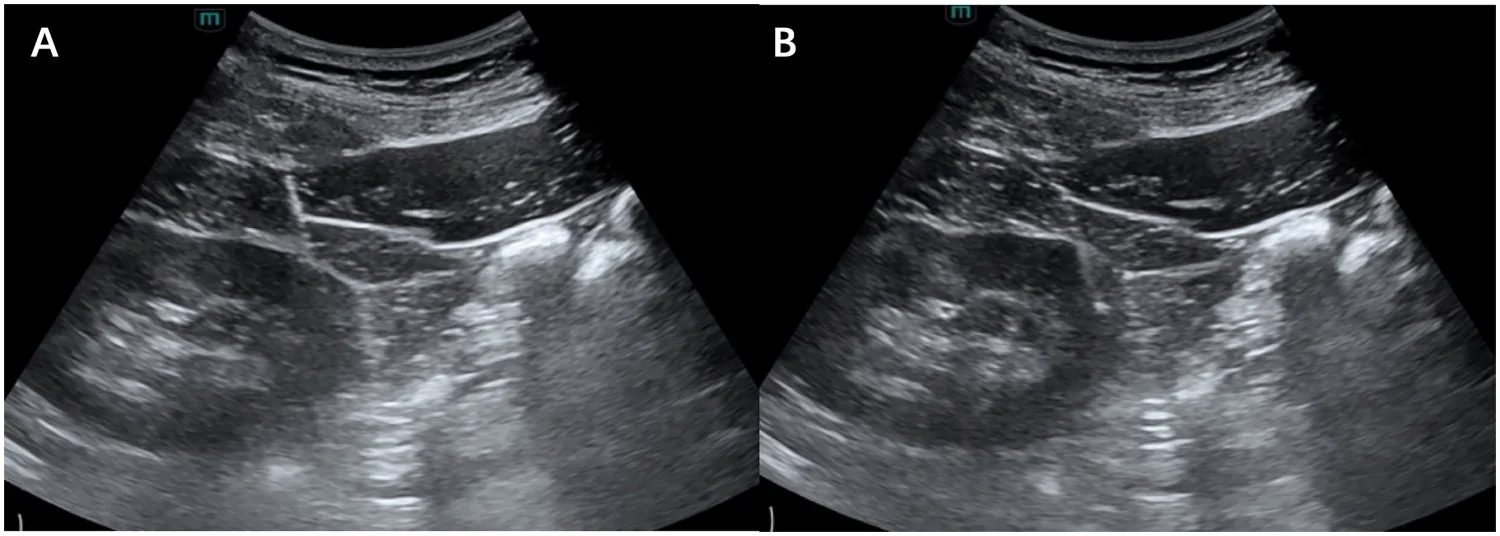
Noncoaxial technique. The lower pole of the kidney is real-time monitored with a longitudinal-sectional probe location; without coaxial needle (A), the automated biopsy needle is advanced to and abut the renal capsule and then fired for tissue sampling (B).

For the noncoaxial technique with longitudinal probe orientation, ultrasonographic localization was performed by placing the probe in a sagittal plane. Under real-time ultrasound guidance, the renal cortical tissue at the renal lower pole was targeted with the needle trajectory directed away from the renal hilum; an 18- or 16-gauge automated core biopsy needle was then advanced to the renal capsule and pressed against it to create a visible indentation without perforation. Prior to needle advancement and activation, the patient was instructed to suspend respiration (Figure 3).

Regardless of which technique or biopsy needle was used, a total of 1–3 needle passes were performed, with a minor angular adjustment made for each pass, to obtain 1–3 renal tissue cores. Each obtained biopsy specimen was immediately evaluated macroscopically by a nephrologist. The decision to perform the second or third needle pass was determined based on the presence of blank attempts (i.e. no tissue retrieval) and the satisfaction with the total length of the specimen assessed visually. Immediate post-biopsy specimen adequacy assessment by a pathologist was not available at our unit.

After the biopsy procedure, the tissue cores were sent immediately to the anatomical pathology laboratory for assessment of specimen adequacy by a pathologist.

At the end of the procedure, all participants underwent immediate ultrasound monitoring for 5–10 min to assess early complications, particularly the presence or progression of perirenal hematoma. Patients were then transferred to the inpatient ward and required to maintain strict supine bed rest for 24 h, with continuous clinical surveillance for 48 h. Routine urinalysis and laboratory blood tests were performed during the monitoring period. Delayed renal ultrasound was performed again post‑procedure if any symptomatic changes or other clinical indications developed. Complications were categorized into minor and major in accordance with guidelines published by the Society of Interventional Radiology (SIR) [9]. Patients were discharged in the absence of any adverse event after 48–72-h hospitalization.

### Data collection

The information about each patient was recorded from covering variables such as age, sex, underlying diseases, blood pressure, pre-procedure laboratory data, renal cortical thickness measured by pre-procedural ultrasound, needle size, number of capsule passes, number of specimen cores, the number of glomeruli in pathological analysis, procedural time (the time from skin penetration to the completion of tissue sampling) the resulting complications (microscopic hematuria, gross hematuria and perinephric hematoma formation).

### Statistical analysis

Statistical analysis was performed using SPSS 23.0.0 for MacOS (IBM, Armonk, New York). Qualitative data were presented as counts and percentages, while quantitative data were summarized as means (± standard deviation) or as medians (and inter-quartile range), depending on the normality of the distribution. Differences were assessed using the Chi-square or Fisher exact test for categorical variables and the Student t-test or Mann–Whitney test for continuous variables. The significance level was set to *p* < 0.05.

## Results

**Figure 4.**
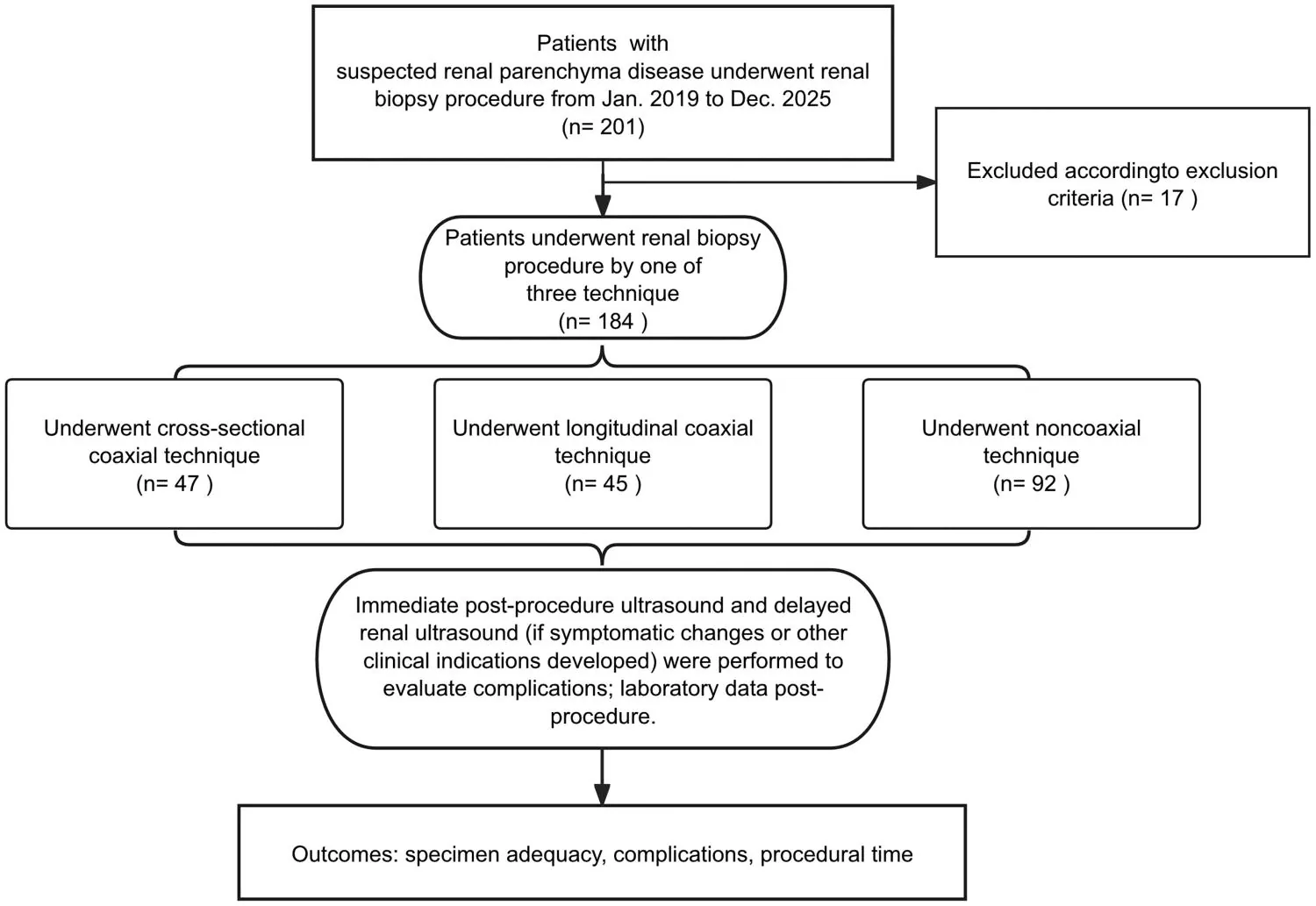
Flow diagram of study population.

During the study period, a total of 201 patients underwent PRB in our center. 184 participants meeting the above criteria were included in the study (Figure 4). renal biopsies were performed in 47 cases using the coaxial technique with a cross-sectional approach (Group A, cross-sectional coaxial group), 45 cases using the coaxial technique with a longitudinal approach (Group B, longitudinal coaxial group), and 92 cases using the non-coaxial technique with a longitudinal approach (Group C, non-coaxial group). Laboratory parameters and demographic data of the patients are summarized in Table 1. Overall, the baseline characteristics of the three groups were comparable, including underlying diseases (incidence of systemic lupus erythematosus and diabetes), pre-procedure laboratory parameters (international normalized ratio, platelet count, and hemoglobin level), and pre-procedure blood pressure. Although statistically significant differences were observed in the selection of the left or right kidney as the biopsy target, these differences have limited clinical significance. There were slight differences in the proportion of 16-gauge and 18-gauge needles among the three groups, but these differences were not statistically significant (*p* = 0.198).

**Table 1.** Baseline characteristics.

	Group A *n* = 47	Group B *n* = 45	Group C *n* = 92	*P*-value
Age	43.7 ± 14.1	38.3 ± 12.9	40.7 ± 14.1	0.185^&^
Sex, *n* (%)				0.544*
Male	20 (42.6)	23 (51.1)	48 (52.2)	
Female	27 (57.4)	22 (48.9)	44 (47.8)	
Underlying systemic lupus erythematosus, *n* (%)	1 (2.1)	3 (6.7)	8 (8.7)	0.268*
Diabetes, *n* (%)	10 (21.3)	12 (26.7)	19 (20.7)	0.716*
Creatinine (umol/L)	107.6 ± 118.1	150.5 ± 204.0	114.6 ± 133.6	0.757^#^
International normalized ratio (INR)	0.93 ± 0.07	0.94 ± 0.09	0.92 ± 0.09	0.590^#^
Platelet count (×10^9^/L)	243.9 ± 69.5	226.5 ± 57.9	224.5 ± 65.2	0.542^#^
Hemoglobin level (g/dL)	12.2 ± 2.3	12.9 ± 2.2	12.7 ± 2.5	0.181^#^
Systolic blood pressure (mmHg)	130.0 ± 18.1	122.6 ± 18.7	128.5 ± 18.0	0.146^#^
Diastolic blood pressure (mmHg)	81.4 ± 10.1	79.9 ± 11.4	81.7 ± 11.0	0.752^#^
Kidney length (mm)	105.8 ± 10.2	107.7 ± 8.2	107.7 ± 9.9	0.434^#^
Renal cortical thickness (mm)	7.6 ± 1.4	7.6 ± 1.4	8.0 ± 1.7	0.304^#^
Left/right kidney, *n* (%)				<0.001*
Left	41 (87.2)	19(42.2)	92 (100.0)	
Right	6 (12.8)	26 (57.8)	0 (0.0)	
Needle size, *n* (%)				
16-gauge	35 (74.5)	31 (68.9)	55 (59.8)	0.198*
18-gauge	12 (25.5)	14 (31.1)	37 (40.2)	

* The Chi-square test was used.

^#^ The Mann–Whitney U test was used.

^&^ The t-test was used.

The outcomes of the three groups are summarized in Table 2. The mean total glomerular yield was 26.9 ± 9.9 (range: 10–51) in the cross-sectional coaxial group, 22.93 ± 10.75 (range: 9–50) in the longitudinal coaxial group, and 20.87 ± 11.50 (range: 8–52) in the non-coaxial group. No statistically significant difference was observed between Groups A and B (*p* = 0.236), whereas statistically significant differences were detected between Groups A and C (*p* < 0.001), as well as between Groups B and C (*p* < 0.001). Technical success was defined as obtaining ≥ 10 glomeruli, a threshold required for histological specimen adequacy [10]. All subjects in the cross-sectional coaxial group had adequate specimens for histopathological evaluation, and only one subject (2.2%, 1/45) in the longitudinal coaxial group did not have an adequate specimen, with the sample containing nine glomeruli. While ten subjects (10.8%, 10/92) did not have an adequate specimen in the non-coaxial group, with the sample containing eight-nine glomeruli. The specimen adequacy was significantly better in the cross-sectional coaxial group than the non-coaxial group (*p* = 0.016), whereas no statistically significant differences were observed between the cross-sectional and longitudinal coaxial groups (*p* = 0.489), nor between the longitudinal coaxial and non-coaxial groups (*p* = 0.101). The number of specimen cores taken to reach an adequate number of glomeruli did not differ significantly between groups (*p* = 0.648, *p* = 1.000).

**Table 2.** Comparison of technical success, procedural time and complications between three groups.

	Group A *n* = 47	Group B *n* = 45	Group C *n* = 92	*P*-value Total	A vs. B	*P*-value B vs. C	A vs. C
Technical success (specimen adequacy, glomeruli ≥ 10), *n* (%)				0.015*	0.489	0.101	0.016
success	47 (100.0)	44(97.8)	82 (89.1)				
unsuccess	0 (0.0)	1 (2.2)	10 (10.8)				
Total glomerular yield	26.90 ± 9.90	22.93 ± 10.75	20.87 ± 10.50	<0.001^#^	0.028^#^	0.236^#^	<0.001^#^
Numbers of specimen cores (median, IQR)	2.0, 2.0–2.0	2.0, 2.0–2.0	2.0, 2.0–2.0	0.648^#^	1.000^#^	0.426^#^	0.471^#^
Number of sampling attempts (median, IQR)	2.0, 2.0–2.0	2.0, 2.0–2.0	2.0, 2.0–2.0	1.000^#^			
Mean number of glomeruli per sampling attempt	13.94 ± 5.21	11.88 ± 5.53	10.67 ± 5.14	0.001^#^	0.048^#^	0.029^#^	<0.001^#^
Procedural time (in mins)	1.45 ± 0.20	2.22 ± 0.35	2.61 ± 0.46	<0.001^#^	<0.001^#^	<0.001^#^	<0.001^#^
Complications, n (%)							
Minor (SIR class A & B)							
Perinephric hematoma	5 (10.6)	11 (24.4)	37 (40.2)	0.001*	0.081*	0.069*	<0.001*
Gross hematuria	7 (14.9)	19 (42.2)	37 (40.2)	0.003*	0.004*	0.823*	0.002*
Microscopic hematuria	35 (74.5)	38 (84.4)	85 (92.4)	0.018*	0.237*	0.149*	0.004*
Major (SIR class C to F)							
Perinephric hematoma requiring embolization	0 (0.0)	0 (0.0)	0 (0.0)				

* The Chi-square test was used.

^#^ The Mann–Whitney U-test was used.

SIR Society of Interventional Radiology.

IQR interquartile range.

The mean number of glomeruli per attempt was calculated as the total glomerular yield divided by the number of sampling attempts. Although the three groups underwent a similar number of sampling attempts (all with a median of 2.0 attempts and interquartile range 2.0-2.0, *p* = 1.000), there was a significant difference in the mean number of glomeruli obtained per attempt (*p* < 0.001), suggesting that the sampling quality of the cross-sectional coaxial group outperformed the longitudinal coaxial group, which in turn outperformed the non-coaxial group (13.94 ± 5.21 vs 11.88 ± 5.53 vs 10.67 ± 5.14, all *p* < 0.001; Table 2).

The mean procedural time, the time from skin puncture to the completion of tissue sampling, differed significantly among the three groups, showing a stepwise increase: the cross-sectional coaxial group < the longitudinal coaxial group < the non-coaxial group (1.45 ± 0.20 min vs 2.22 ± 0.35 min vs 2.61 ± 0.46 min; all *p* < 0.001; Table 2).

No major complications (perinephric hematoma requiring embolization) occurred in any of the three groups. However, statistically significant differences were observed among the three groups with regard to the three minor complications: perinephric hematoma, gross hematuria, and microscopic hematuria. Bleeding complications were more prevalent in the non-coaxial group than in the cross-sectional coaxial group, with significant differences observed for perinephric hematoma (*p* < 0.001), gross hematuria (*p* = 0.002), and microscopic hematuria (*p* = 0.004). A significantly higher incidence of gross hematuria was also noted in the cross-sectional coaxial group compared to the longitudinal coaxial group (*p* = 0.004). No significant differences in perinephric hematoma (*p* = 0.081) and microscopic hematuria (*p* = 0.237) were found between the two coaxial groups. No significant differences in these three complications were found between the longitudinal coaxial group and the non-coaxial group (Table 2).

None of the three groups required interventional treatment for hemorrhagic complications. No prolonged hospital stay due to complications was observed. Accordingly, no major complications (defined as SIR class C to F) occurred in this study cohort.

## Discussion

Literature on either the coaxial technique [6,11,12] or the cross-sectional approach [7,8] for native kidney biopsy in evaluating renal parenchymal disease is limited, and there is arguably an absence of publications specifically addressing the coaxial cross-sectional approach. The advantages of the cross-sectional approach in native kidney biopsy remain undefined. This current study compared the use of the three techniques (coaxial cross-sectional technique, coaxial longitudinal technique, and a conventional non-coaxial technique) for percutaneous US-guided biopsy of renal parenchyma, in terms of total glomerular yield, specimen adequacy, complications, and procedural time. This study demonstrated that renal biopsy *via* the coaxial cross-sectional technique yielded a higher glomerular count, a shorter procedural time, improved specimen adequacy, and a lower incidence of complications compared with the coaxial longitudinal and non-coaxial techniques.

The advantages of the coaxial technique are that multiple tissue cores can be obtained *via* a single puncture of the renal capsule, thereby increasing the glomerular yield. In the present study, the coaxial group required a mean of only 1.0 coaxial needle passes, whereas the non-coaxial group had a mean of 2.0 core needle passes for the biopsy procedure. A key disadvantage of the coaxial technique is that a larger-gauge (15/17-gauge) outer cannula is required to obtain a 16/18-gauge core biopsy sample, which may potentially increase the risk of renal parenchymal injury. Babaei Jandaghi et al. [6] performed a randomized controlled trial on 166 adult native kidney biopsies, noting that the coaxial group had a higher glomerular yield, shorter procedural time, and lower complication rate (10.8% vs 24.1%). Similar findings were yielded in the present study upon comparison of the coaxial and non-coaxial cohorts, with the coaxial technique group exhibiting a higher technical success rate and a lower incidence of hemorrhagic complications relative to the non-coaxial counterpart. A retrospective analysis of 239 renal biopsy specimens revealed that the coaxial technique mitigates mild to moderate adverse events associated with PRB, yet exerts no notable influence on the incidence of severe adverse events (coaxial cohort: 4/143 cases, 2.8%; non-coaxial cohort: 3/94 cases, 3.2%; *p* = 0.567) [11]. By comparison, a separate retrospective investigation by Hatfield et al. encompassing 1,060 solid organ biopsies (including renal and hepatic procedures) identified no statistically significant discrepancy in complication rates between the coaxial and non-coaxial approaches [13]. Notably, however, this study lacked a stratified subgroup analysis focusing exclusively on renal biopsy cases. In certain studies [2], lower complication rate may be attributable to both fewer capsular punctures required and biopsy tract plugging with absorbable gelatin sponge when using the coaxial method.

Traditionally, biopsy fragments are obtained at the lower pole of the kidney, in the sagittal axis of the unit, either in the craniocaudal or caudocranial direction. Other authors have used the axial plane of the lower pole as the site of biopsy [7,14]. In this study, biopsy fragments obtained from the renal parenchyma of the mid-to-lower pole of the native kidney in the axial plane achieved a significantly higher technical success and a better sampling quality suggested by the mean number of glomeruli per attempt (*p* < 0.001), since this region tends to offer the greatest transverse diameter of cortex for core optimization. Notably, the incidence of hemorrhagic complications in the coaxial cross-sectional group was statistically significantly lower compared with the other two groups, which demonstrates the synergistic benefits derived from the integration of the coaxial and transverse modalities.

Surasit Akkakrisee et al. [8] measured the area of the target zone of the renal cortex for biopsy, revealing that the cross-sectional approach tends to have a larger possible area for biopsy compared to that of the longitudinal approach. In our practice, this advantage has proven to be effective, and we have further identified more strengths of the cross-sectional approach. The cross-sectional approach tends to enable a more favorable and stable angle (a larger angle than that with the sagittal approach), facilitating easier needle insertion and effectively reducing the risk of needle slippage. Meanwhile, it shortens the needle travel distance through the perirenal fat and renal parenchyma, which not only reduces the overall procedural time but also lowers the risk of renal parenchymal injury caused by an excessively long needle travel path; additionally, the larger needle insertion angle in this approach allows for more stable monitoring of the needle tip position. In contrast, under sagittal guidance, the puncture needle is mostly inserted obliquely with a relatively smaller angle to the renal surface, which not only increases the risk of needle slippage and prolongs the needle travel distance but also results in more significant traction injury to the renal parenchyma.

All three techniques employed in the present study emphasize that the outer sheath of the coaxial needle (for the coaxial technique) or the biopsy needle (for the non-coaxial technique) should be pressed gently against the renal capsule at the target site to create a visible indentation without perforation. This maneuver is combined with patient breath-holding to prevent needle slippage upon biopsy needle activation, which may otherwise result in sampling failure. This standardized procedural step is likely one of the reasons why the overall technical success rate in our study (89.1-100.0%) is higher than the rates reported in other relevant studies (62.9-85.7%) [8]. In particular, in the cross-sectional approach, the mid-to-lower pole renal parenchyma can all serve as a puncture target zone, thus conferring a higher tolerance for renal displacement caused by breathing movement. And even when the patient is breathing, the angle of the biopsy needle does not move as much as it does when the longitudinal technique is used.

Although the selection of the left or right kidney as the biopsy target is influenced by multiple factors, in the non-coaxial technique group, the radiologists and equipment at our center were generally positioned on the side of the patient’s left kidney. The convenience of operating on the adjacent side led to a significant difference between this group and the other two groups. However, it is generally accepted that there are no intrinsic differences between the left and right kidneys in terms of sampling quality and bleeding risk, and the selection of the renal side is determined by anatomical assessment and procedural convenience [15].

While our analysis focused on adult patients, the utility of coaxial techniques in optimizing renal biopsy adequacy and safety—evidenced by Fung et al. in their pediatric cohort (100% specimen adequacy, 4.5% hemorrhagic complications, and higher glomerular yield with coaxial vs. non-coaxial methods) [2]—suggests potential translatability of our cross-sectional coaxial approach to pediatric populations. The pediatric population poses greater challenges to the efficiency and safety of PRB due to smaller renal size, thinner renal cortex, and more compact intra-abdominal anatomy. The transverse imaging plane enables a gentler puncture angle and a shorter needle path, which may reduce traction and injury to the thin renal cortex in children; moreover, given poor patient cooperation and limited sedation time in pediatric PRB, the high sampling efficiency of the cross-sectional coaxial technique can shorten procedural duration, minimize sedation-related risks. These theoretical advantages support the potential value of the cross-sectional coaxial technique in the pediatric setting, particularly in selected patients with favorable body habitus and renal anatomy, although further clinical validation is warranted.

## Limitations

There are several limitations to our study. It is a retrospective review of a single tertiary referral center with a relatively small sample size, therefore limiting the generalizability. The choice of technique depended on the operator’s preference, which might have biased the results. All three groups used 18-gauge and 16-gauge core biopsy needles. Although literature reports that needle gauge affects specimen adequacy and bleeding complication [16–18], we could not conduct a subgroup analysis for this. Further study is needed to compare the performance of 16-gauge needles and 18-gauge needles with the coaxial cross-sectional technique. Subgroup analyses were not performed for patients with different body mass indexes (BMIs) or different types of renal diseases. Future randomized trial of multicenter with larger sample sizes could provide the statistical power required to confirm the trends observed in this study and further refine the selection of biopsy techniques based on patient-specific anatomical and physiological factors.

## Conclusion

This single-center retrospective investigation demonstrates that, for ultrasound-guided renal biopsy, the coaxial technique with a cross-sectional probe location was independently linked to a higher technical success rate relative to both the coaxial longitudinal approach and the non-coaxial longitudinal approach, alongside a reduced incidence of hemorrhagic complications. Additionally, this coaxial cross-sectional method was associated with a markedly shorter procedural duration compared with the other two techniques, thereby confirming its superior procedural efficiency. Nevertheless, given the study’s limitations related to sample size and underpowered subgroup analyses, these findings require cautious interpretation. Larger-scale, randomized multicenter studies are therefore warranted to verify the potential benefits of the coaxial cross-sectional technique.

## Data Availability

The datasets used in this work are accessible upon reasonable request from the corresponding author.
